# Correction: Earl et al. Somatic Mutation Profiling in the Liquid Biopsy and Clinical Analysis of Hereditary and Familial Pancreatic Cancer Cases Reveals KRAS Negativity and a Longer Overall Survival. *Cancers* 2021, *13*, 1612

**DOI:** 10.3390/cancers13153687

**Published:** 2021-07-22

**Authors:** Julie Earl, Emma Barreto, María E. Castillo, Raquel Fuentes, Mercedes Rodríguez-Garrote, Reyes Ferreiro, Pablo Reguera, Gloria Muñoz, David Garcia-Seisdedos, Jorge Villalón López, Bruno Sainz, Nuria Malats, Alfredo Carrato

**Affiliations:** 1 Molecular Epidemiology and Predictive Tumor Markers Group, Ramón y Cajal Health Research Institute (IRYCIS), Carretera Colmenar Km 9100, 28034 Madrid, Spain; emma.barreto@salud.madrid.org (E.B.); marien.castillo@salud.madrid.org (M.E.C.); rfuentes@salud.madrid.org (R.F.); mercedes.rodriguez@salud.madrid.org (M.R.-G.); reyes-ferreiro@hotmail.com (R.F.); pablo.reguera@salud.madrid.org (P.R.); jorge.villalon@salud.madrid.org (J.V.L.); alfredo.carrato@salud.madrid.org (A.C.); 2 Biomedical Research Network in Cancer (CIBERONC), C/Monforte de Lemos 3-5. Pabellón 11, 28029 Madrid, Spain; nmalats@cnio.es; 3 Translational Genomics Core Facility, Ramón y Cajal Health Research Institute (IRYCIS), 28034 Madrid, Spain; mariagloria.munoz@salud.madrid.org (G.M.); dgarcia@ebi.ac.uk (D.G.-S.); 4 Department of Biochemistry, Universidad Autónoma de Madrid (UAM), Ramón y Cajal Health Research Institute (IRYCIS), 28034 Madrid, Spain; bsainz@iib.uam.es; 5 Instituto de Investigaciones Biomédicas “Alberto Sols” (IIBM), CSIC-UAM, C/Arzobispo Morcillo, 4, 28029 Madrid, Spain; 6 Cancer Stem Cell and Fibroinflammatory Group, Chronic Diseases and Cancer, Area 3-IRYCIS, 28029 Madrid, Spain; 7 Genetic and Molecular Epidemiology Group, Spanish National Cancer Research Centre (CNIO), 28029 Madrid, Spain; 8 Department of Medicine and Medical Specialties, Medicine Faculty, Alcala University, Plaza de San Diego, s/n, 28801 Alcalá de Henares, Spain

The authors wish to make the following corrections to this paper [1]: In the published version, Figure 4 appeared as a duplication of Figure 1b. Furthermore, the legend of Figure 2 has been corrected to accurately reflect the data shown.

The correct version of Figure 2 is as follows:

**Figure 2:**
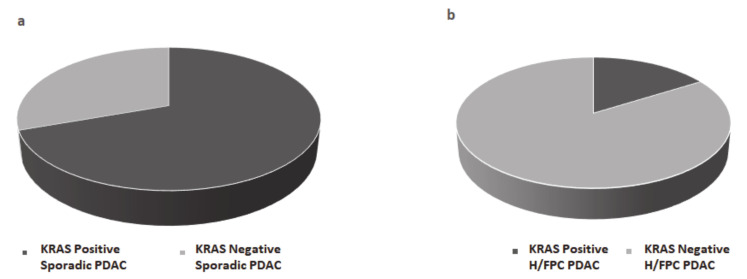
*KRAS* mutation status was determined in plasma from (**a**) sporadic PDAC cases (**b**) hereditary or familial PDAC (H/FPC) cases via BEAMing and mutant *KRAS* was more frequently detected in sporadic cases compared to H/FPC cases. BEAMing was performed using cfDNA isolated from 1 mL of plasma from 54 PDAC cases (31 familial cases and 23 sporadic cases). The frequency of mutant *KRAS* was 70% in sporadic cases and 16% in familial cases, which was statistically significant (*p* ≤ 0.001).

The correct version of Figure 4 is as follows:

**Figure 4:**
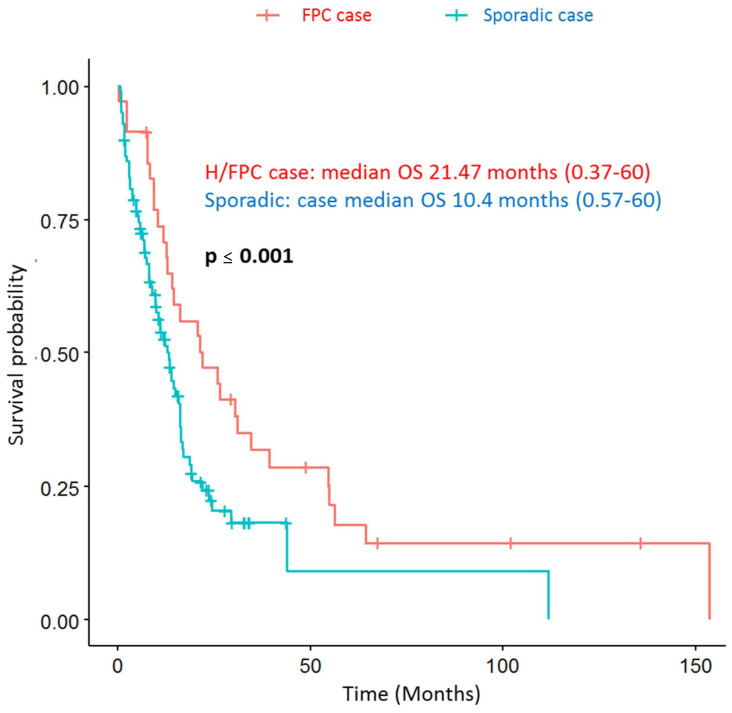
Hereditary or familial PDAC cases have a longer overall survival (OS) compared to sporadic cases.

We stress that these errors were purely due to human error and oversight; the corrections made do not affect or change the written portion of the figure legend, the interpretation of the results, or the final conclusions of this manuscript. The manuscript will be updated. The authors would like to apologize for any inconvenience caused. All changes have been reviewed and verified by the Academic Editors.
