# Supplementary material for: Somatic Mutation Profiling in the Liquid Biopsy and Clinical Analysis of Hereditary and Familial Pancreatic Cancer Cases Reveals KRAS Negativity and a Longer Overall Survival
Source: Cancers (Basel). 2021 Mar 31;13(7):1612. doi: 10.3390/cancers13071612 (PMC8038004; doi:10.3390/cancers13071612)
Supplement: Supplementary file 1 [file cancers-13-01612-s001.zip › cancers-1161334 Supplementary Materials.docx]

Article

Supplementary Materials: Somatic Mutation Profiling in the Liquid Biopsy and Clinical Analysis of Hereditary and Familial Pancreatic Cancer Cases Reveals KRAS Negativity and a Longer Overall Survival

Julie Earl, Emma Barreto, María E. Castillo, Raquel Fuentes, Mercedes Rodríguez-Garrote, Reyes Ferreiro, Pablo Reguera, Gloria Muñoz, David Garcia-Seisdedos, Jorge Villalón López, Bruno Sainz, Jr., Nuria Malats and Alfredo Carrato

**Data S1.** Analysis pipeline of for the identification of pathogenic somatic variants in cfDNA using the TruSight 15 kit and The BaseSpace Variant Interpreter tool.

The TruSight Tumor 15 library preparation assay follows a tiling method that uses 2 oligo pools for multiplex PCR, which allows the coverage of larger DNA regions, produces higher coverage uniformity and reduces the presence of primer dimers. This results in a high accuracy and sensitivity. The TruSight Tumor 15 workflow evaluates short regions of amplified DNA in the 2 oligo sequencing pools (Mix A and Mix B) to analyze targeted regions. These sequences are aligned to the hg19 reference genome for coordinates and chromosome mapping. The variant scores are computed using a Poisson model that filters variants with a quality score below Q30 and only calls variants for bases that have a coverage depth of ≥500.

Variants are called for Mix A and Mix B separately and are only reported in the variant file if they have a depth of ≥500 and a variant frequency of 2.6% or greater. The Exclusion criteria for variants are as follows:

- The variant frequency is near the signal noise level, between 1% and 2.6%
- The depth is <500
- Significant strand bias is detected, which can be a sequencing-specific error

Variants are annotated in the vcf file with:

- Gene name
- Nucleotide change
- Transcript
- Amino acid change
- Consequence

**Table S2.** Summary of the Cox Proportional-Hazards Model analysis with the corresponding odds ratios showing the effect size of the covariates included in the survival analysis.

|  | **exp(coef)** | **se(coef)** | **exp(-coef)** | **lower .95** | **upper .95** | **z** | **Pr(>\|z\|)** |
| --- | --- | --- | --- | --- | --- | --- | --- |
| **Low cfDNA level** | 0.562211 | 0.3226 | 1.7787 | 0.3226 | 0.9798 | −2.032 | 0.04216* |
| **Medium cfDNA level** | 0.523617 | 0.31309 | 1.9098 | 0.31309 | 0.8757 | −2.466 | 0.01367* |
| **High cfDNA level** | 1.7787 | 0.283409 | 0.5622 | 1.02062 | 3.0998 | 2.032 | 0.04216* |
| **Sporadic PDAC** | 2.413846 | 0.256857 | 0.41428 | 1.45906 | 3.9934 | 3.431 | 6.02E-04** |

** *p* ≤ 0.01; * *p* ≤ 0.05.

**Figure S1.** Comparative cfDNA extraction from 1ml of plasma of 3 samples using the (**a**) QIAamp circulating nucleic acid kit and (**b**) Maxwell^®^ RSC Instrument.

A higher overall concentration of cfDNA of approximately 180 bp (dashed arrow) was obtained using the Qiagen kit, although with higher levels of contaminating genomic DNA of >1500 bp from blood cells (solid arrow).

|   **a** | 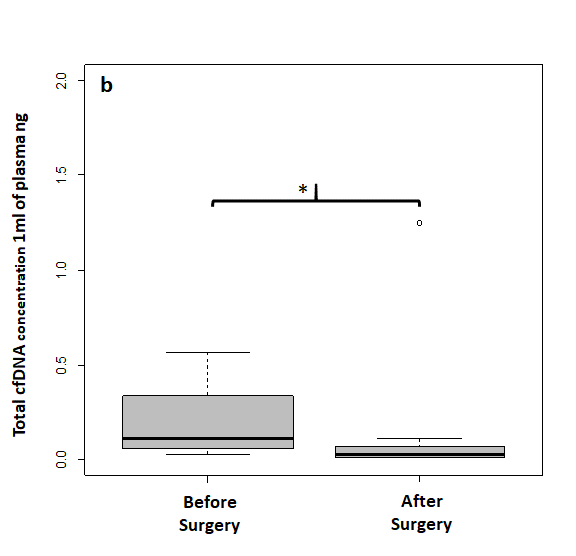  **b** |
| --- | --- |

**Figure S2.** Correlation of circulating free DNA (cfDNA) levels with clinical parameters (disease stage, disease status and surgery). (**a**) The concentration of cfDNA in plasma differentiates between cancer and non-cancer cases and correlates with PDAC stage. The median cfDNA level in resectable cases was 0.0575 ng (0.0050–2.0000), 0.0675 ng (0.0350–2 ng) in locally advanced and 0.0700 ng (0.005-4) in metastatic cases. Significantly higher levels of cfDNA were found in PDAC cases of all stages and patients with pancreatic cysts compared to healthy controls. Furthermore, patients with locally advanced and metastatic disease had significantly higher cfDNA levels compared to patients with pancreatic cysts. (**b**) cfDNA levels are significantly reduced in plasma after a surgical resection of the primary tumor. There was a significant reduction in the cfDNA level from 0.11 ng (0.025–5.5 ng) before surgery to 0.025 ng (0.01–1.25 ng) after surgery.
